# Olfactory loss is a predisposing factor for depression, while olfactory enrichment is an effective treatment for depression

**DOI:** 10.3389/fnins.2022.1013363

**Published:** 2022-09-28

**Authors:** Michael Leon, Cynthia C. Woo

**Affiliations:** ^1^Department of Neurobiology and Behavior, University of California, Irvine, Irvine, CA, United States; ^2^Center for the Neurobiology of Learning and Memory, Institute for Memory Impairments and Neurological Disorders, University of California, Irvine, Irvine, CA, United States

**Keywords:** olfaction, depression, brain reserve, neurological disorders, brain stimulation

## Abstract

The loss of olfactory stimulation correlates well with at least 68 widely differing neurological disorders, including depression, and we raise the possibility that this relationship may be causal. That is, it seems possible that olfactory loss makes the brain vulnerable to expressing the symptoms of these neurological disorders, while daily olfactory enrichment may decrease the risk of expressing these symptoms. This situation resembles the cognitive reserve that is thought to protect people with Alzheimer’s neuropathology from expressing the functional deficit in memory through the cumulative effect of intellectual stimulation. These relationships also resemble the functional response of animal models of human neurological disorders to environmental enrichment, wherein the animals continue to have the induced neuropathology, but do not express the symptoms as they do in a standard environment with restricted sensorimotor stimulation.

## Introduction

### Epidemiology of depression

Neurological disorders are the greatest cause of disability and the second greatest cause of deaths around the world (GBD 2015 Neurology Collaborators, 2016). One in two women and one in three men will develop dementia, stroke, or Parkinson’s disease during their lifetime (Licher et al., 2019). Depression is characterized by feelings of sadness and hopelessness, and is the leading neurological disorder in disability-adjusted life years, (i.e., the sum of years lost due to premature mortality plus the years with a disability). Around 350 million people are affected by depression around the world (Lopez and Murray, 1998; GBD 2015 Neurology Collaborators, 2016). The lifetime risk of depression in the U.S. is about 20% (Hasin et al., 2018), and in other affluent countries, 40% of women and 30% of men have been found to have this disorder before they are 65 years old (Kruijshaar et al., 2005), making it a major medical issue. The existing pharmaceutical treatments for this serious emotional problem range from somewhat effective to largely ineffective (Rush et al., 2006; Ioannidis, 2008; Cipriani et al., 2018). Moreover, the drugs come with a wide range of side effects, including changes in bodyweight, sleep pattern, and libido (Ramic et al., 2020).

### Drug treatment for depression

While most of the drugs used to treat depression are specific serotonin reuptake inhibitors (SSRIs), it is unlikely that the efficacy of increasing this or other monoamines improves the emotional status of people with depression through the mechanism of treating a monoamine deficiency (Boku et al., 2018). Rather, it is more likely that the effects of these drugs are mediated by their ability to increase levels of brain-derived neurotropic factor (BDNF). Increased BDNF has a salutary effect on keeping hippocampal neurons alive under conditions of chronic stress, which is often involved in triggering a depressive episode (Björkholm and Monteggia, 2016; Tafet and Nemeroff, 2016). It is the neural repopulation of this brain area that seems to be critical in recovering from the disorder (Boku et al., 2018).

Two new antidepressant drugs have been approved recently by the FDA and they use very different mechanisms of action to improve depression symptoms. Ketamine in a racemic mixture of R-ketamine and S-ketamine has been in use as an anesthetic, is administered as an infusion (i.v.), and also has antidepressant action when used in lower doses and acts rapidly compared to other antidepressants (Covvey et al., 2012). Esketamine (S-ketamine) alone, administered as a nasal spray, has also been shown to reduce depression symptoms, also with a rapid onset of action (Dean et al., 2021; Doty et al., 2021). However, for both drugs, there is very low−certainty evidence provided for their efficacious use (Dean et al., 2021; Doty et al., 2021). Both versions of ketamine seem to have their mechanism of action as an antagonist on the NMDA glutamate receptor.

Another drug with a more convenient, oral method of administration also has been approved recently for use in treatment-resistant major depression and it also seems to have a rapid action to suppress depression symptoms. Called Auvelity, it contains dextromethorphan, which is an anti-tussive that is a non-competitive antagonist for the NMDA receptor and to the sigma opioid receptor, and bupropion, an antidepressant on its own that increases synaptic norepinephrine and dopamine by decreasing their reuptake (Stahl et al., 2004; Iosifescu et al., 2022).

### Electrical stimulation as a treatment for depression

Only 40–60% of patients with major depressive disorder are successfully treated with pharmaceuticals (Rush et al., 2006; Malhi et al., 2020). One alternative for drug treatment-resistant patients is the use of electrical stimulation. Electrical brain stimulation in the form of electroconvulsive therapy can be an effective treatment for depression and has the advantage of being fast-acting, compared to the weeks required before SSRIs become effective (Li et al., 2021). The rapidity with which this treatment works allows it to be used by people who are severely depressed and who are considering suicide. In this method of brain activation, electrical stimulation is delivered to the skull and a *grand mal* seizure is induced in the patient. This approach is often successful, with 50–60% of drug treatment-resistant patients coming into remission with this therapy (Mutz et al., 2019). One of the adverse side effects, however, include the loss of recent memories (Espinoza and Kellner, 2022).

### Transcranial stimulation as a treatment for depression

Transcranial magnetic stimulation uses electromagnetic stimulation to treat major depressive disorder by activating the dorsolateral prefrontal cortex. Compared to electrical stimulation, a benefit of this method is greater spatial and temporal control, without provoking seizures (Rizvi and Khan, 2019). While different systems and protocols produce different outcomes, a typical finding is that after 5 sessions per week for 4 weeks, followed by a continuation phase of twice-weekly treatment for an additional 12 weeks, the remission rates for drug treatment-resistant patients were 32.6% for transcranial magnetic therapy vs. 14.6% for sham treatment (Perera et al., 2016). Although moderately successful for some drug treatment-resistant patients, it requires multiple medical visits which can be inconvenient, expensive, and time-consuming.

### Deep brain stimulation as a treatment for depression

Another treatment method is deep brain stimulation (DBS), which involves implanting electrodes in the brain and using electrical stimulation to regulate brain activity. DBS has been used in a variety of brain regions to treat drug treatment-resistant depression and has had some level of success (Dandekar et al., 2018; Aibar-Durán et al., 2022). However, this treatment incurs high costs and lacks consistent positive outcomes.

### Animal models of depression

Ideally, the treatment for depression should be highly effective for all levels of the disorder, inexpensive, effortless, and without significant side effects. A different approach has been developed using animal models of depression, which mimic both the etiology of depression and the human-like depression symptoms (McKinney and Bunney, 1969). Indeed, depression-like symptoms resembling that seen in the human response to chronic stress can be induced in rats and mice following chronic isolation, pain, learned helplessness, social defeat, minor stressors, maternal deprivation, striatal intracerebral hemorrhage, forced swimming, restraint, olfactory bulb removal, and tail suspension (Kelly et al., 1997; Veena et al., 2009; Richter et al., 2013; Gong et al., 2018; Seong et al., 2018; Hao et al., 2019; Wang et al., 2019; Sparling et al., 2020; Borba et al., 2021; Cordner et al., 2021; Ramírez-Rodríguez et al., 2021; Réus et al., 2021; Taheri Zadeh et al., 2021; Kimura et al., 2022). Moreover, the therapies used to treat humans with depression are also effective in reversing induced depression in lab animals, including antidepressants, electroconvulsive seizure therapy, and deep brain stimulation (Jeannotte et al., 2009; Falowski et al., 2011; Tizabi et al., 2012; Belujon and Grace, 2014; Song and Kim, 2021).

## Environmental enrichment

### Environmental enrichment improves cognitive ability, emotional status, and the human-like symptoms of many neurological disorders in lab animals

This phenomenon was initially described by Hebb (1947), who found neurobehavioral improvements in rats that he kept uncaged at his home, compared to the rats kept in small box cages in the lab. Other researchers then systematized the experimental paradigm to keep the enriched rats in a large cage with other conspecifics, an exercise wheel, and multiple children’s toys that were changed on a regular basis (Rosenzweig et al., 1962). Control rats kept in standard cages either had a single conspecific or no conspecifics, and no additional features. Compared to controls, the brains of the enriched rats were much larger and more complex and had higher levels of neurotransmitters and trophic factors. Enriched rats also exhibited improved cognitive ability, emotional status, and motor ability. A large body of work has followed this landmark study, and impressively, in animal models of many other human neurological disorders, environmental enrichment greatly ameliorated or completely eliminated symptoms of these and other disorders: autism, stroke, seizures, traumatic brain injury, ADHD, prenatal alcohol syndrome, lead exposure, multiple sclerosis, addiction, schizophrenia, epilepsy, Alzheimer’s Huntington’s disease, Parkinson’s disease, Down syndrome, fragile X syndrome, Rett syndrome, and Potocki-Lupski syndrome (Nithianantharajah and Hannan, 2009; Hannan, 2014).

### In rodents, environmental enrichment improves the neural and cognitive loss normally experienced with aging

A loss of hippocampal neurons is involved in the gradual memory loss experienced with normal aging, and a highly specific form of environmental enrichment can reverse such neural and cognitive changes. Specifically, exposing mice to olfactory enrichment, which involved the daily exposure to multiple diverse odorants, restored the hippocampal neural population and improved cognition in older mice. Veyrac et al. (2009) showed that in mice, olfactory enrichment alone both improved memory and increased brain neurogenesis. They further showed that novelty was the critical element in this kind of stimulation, as exposure to multiple odorants individually resulted in these changes, while exposure to odorant mixtures did not. Rusznák et al. (2018) also showed that exposure to various essential oils alone for 30 min/day over 3 months induced neurogenesis in both the olfactory bulb and the hippocampus, and the neuronal number in those regions increased to a level comparable to what they found in mice housed in an enriched environment. Finally, Zhang et al. (2022) found that olfactory enrichment for 21 days, initiated prior to anesthesia/laparotomy surgery, normalized post-anesthesia/surgery olfactory and cognitive functioning, which were diminished in animals not receiving the enrichment.

### Environmental enrichment improves depression symptoms in animal models

Animal models of depression show improvement in behavioral and biochemical depression-like symptoms following environmental enrichment (Ilin and Richter-Levin, 2009; Veena et al., 2009; Richter et al., 2013; Gong et al., 2018; Seong et al., 2018; Hao et al., 2019; Wang et al., 2019; Brenes et al., 2020; Moradi-Kor et al., 2020; Sparling et al., 2020; Borba et al., 2021; Cordner et al., 2021; Huang et al., 2021; Ramírez-Rodríguez et al., 2021; Taheri Zadeh et al., 2021; Kimura et al., 2022). For example, Jha et al. (2011) found that environmental enrichment treatment in a mouse model of depression not only reversed depression-like behavior, it also restored the reductions in neurogenesis and BDNF levels in the hippocampus.

### Olfactory system connectivity is ideally situated to improve cognition and depression in humans

The olfactory system is the only sensory system that has direct projections to the brain’s cognitive and emotional control areas—the hippocampal-amygdala complex (Haberly and Price, 1977; Zhou et al., 2019; Noto et al., 2021), while the other sensory systems have indirect connections via the thalamus. The unique access of the olfactory system to the cognitive and emotional systems may allow highly specific activation of these systems that could improve cognitive and emotional symptoms.

### Loss of olfactory ability induces deterioration of cognitive brain areas

Olfactory deterioration occurs just before the deterioration of cognitive ability in older adults (Doty et al., 1984; Schaie et al., 2004; Sanna et al., 2021). In addition, the loss or compromise of the olfactory system results in a significant volume loss of both gray matter and white matter in human brains (Bitter et al., 2010a,b; Segura et al., 2013; Yao et al., 2014; Kollndorfer et al., 2015). Even a chronic stuffed nose resulted in the deterioration of gray matter density in the adult brain (Han et al., 2017). Olfactory dysfunction also predicts cognitive dysfunction in human adults (Choi et al., 2018). Moreover, a degradation of olfactory ability predicts an elevated risk of mild cognitive impairment (MCI) and it also predicts which individuals with MCI will develop Alzheimer’s disease (Peters et al., 2003; Adams et al., 2018).

### Increasing olfactory stimulation restores olfactory function

Olfactory enrichment has been shown to improve olfactory function in humans who have experienced olfactory loss due to a variety of problems, such as post-infectious olfactory dysfunction, head trauma, Parkinson’s, and aging (Hummel et al., 2009; Haehner et al., 2013; Konstantinidis et al., 2013; Damm et al., 2014; Geißler et al., 2014; Patel et al., 2017).

### Environmental enrichment, including olfactory enrichment, improves autism symptoms

Environmental enrichment using olfactory enrichment as a central component reduces the symptoms of autism significantly within 6 months in 42% of the children, compared to 7% in standard care, improves IQ by 8 points, compared to no improvement in standard care, improves communication by over 200%, compared to 8% in standard care, and reverses the diagnosis of 21% of the enriched children with classic autism, compared to 0% in standard care (Woo and Leon, 2013; Woo et al., 2015).

This autism treatment is now available with an online algorithm that individualizes the enrichment exercises according to the child’s current symptoms. When we looked at the outcomes of over 1,000 children along the entire autism spectrum (Aronoff et al., 2016), we found that these children did even better than those in the original clinical trials, with a large effect size of 1.85. Not only did the core symptoms of autism improve, but the co-morbid symptoms, also improved: sensory processing, self-awareness, sleeping, communication, eating, motor skills, learning, memory, anxiety, attention and mood, including depression symptoms.

## Olfaction is associated with depression

### Olfactory impairment and depression

Many studies have shown that olfactory impairment is associated with depression in human adults (Atanasova et al., 2008; Croy et al., 2014; Kohli et al., 2016; Croy and Hummel, 2017; Taalman et al., 2017; Rochet et al., 2018; Rottstaedt et al., 2018; Wegener et al., 2018; Pabel et al., 2020; Qazi et al., 2020; Wang et al., 2020; Athanassi et al., 2021; Kim and Bae, 2022; Liu et al., 2022; Sabiniewicz et al., 2022). Moreover, olfactory dysfunction precedes and predicts the development of depression in older adults (Eliyan et al., 2021). Additionally, individuals with congenital anosmia have elevated Beck Depression Inventory scores relative to nornosmic controls (Croy et al., 2012) and higher depression scores are observed in people with either congenital anosmia or acquired anosmia relative to nornosmic controls (Lemogne et al., 2015; Kohli et al., 2016).

In a longitudinal study, adults who had olfactory dysfunction were more likely to develop depression symptoms after 5–10 years than adults who had no olfactory loss at baseline. On the other hand, those adults who had depression symptoms at baseline were not more likely to develop olfactory dysfunction. These data resemble those in which olfactory dysfunction occurs well before the motor or cognitive problems emerge in Parkinson’s and Alzheimer’s diseases (Sohrabi et al., 2012; Devanand, 2016; Fullard et al., 2017). Moreover, the severity of the olfactory dysfunction predicts the severity of depression (Kohli et al., 2016) and the size of the olfactory bulb predicts both the severity of depression and the probability of therapeutic success (Negoias et al., 2016). It is also the case that in lab animals, removal of the olfactory bulb and the consequent loss of olfaction induces a depression-like state that has been used as a model of the disorder (Kelly et al., 1997), the symptoms of which can be ameliorated by anti-depressant drugs (Jarosik et al., 2007).

### Can olfactory enrichment also help to treat depression?

Olfactory enrichment modifies brain structures and improves cognitive and emotional status. Al Aïn et al. (2019) investigated the effects of 6 weeks of olfactory enrichment in healthy young adults and found that the enrichment led to increased cortical thickness in the inferior frontal gyrus, the bilateral fusiform gyrus and the entorhinal cortex compared to visual-training controls. Gellrich et al. (2017) found that olfactory enrichment of people with olfactory deficiencies increased gray matter volume in the hippocampus and the thalamus, changes that could underly the ability of olfactory enrichment to improve cognitive and emotional status in older adults. Han et al. (2021) found that individuals using olfactory enrichment with 4 odorants for 7 months had improved odor identification and larger gray matter volume in the bilateral cerebellum, bilateral thalamus, precentral gyrus, gyrus rectus, and medial orbitofrontal cortex.

Olfactory enrichment initiated after the onset of neurological symptom also can ameliorate those symptoms. Haehner et al. (2013) showed that patients with Parkinson’s disease improved their verbal fluency after olfactory enrichment. Wegener et al. (2018) provided olfactory enrichment to older adults using 4 essential-oil odorants twice a day for 5 months. Controls solved daily Sudoku puzzles during that time. The olfactory-enriched group had a significant improvement of olfactory function, improved verbal function, and decreased depression symptoms.

Cha et al. (2022) exposed 34 older adults with dementia (but with a Mini-Mental State Examination score of at least 10) to 40 odorants twice a day for 15 days. The control group consisted of 31 individuals with dementia that received no such stimulation; there were no initial differences between groups. Their results were remarkable, as the olfactory-enriched group showed highly significant improvements in memory, olfactory identification, depression symptoms, attention, and language skills. In detail, they found that olfactory-enriched individuals had an improvement in olfactory identification, while controls did not, as assessed by the Olfactory Identification Test (*p* < 0.001). The Verbal Fluency Test to assess semantic memory also showed significant improvements for the enriched group relative to the controls (*p* = 0.001). Similarly, the Boston Naming Test to assess language function revealed a significant improvement in the enriched subjects relative to controls (*p* = 0.001). The Word-List Memory Test to assess attention and working memory, the Word-List Recall Test to assess verbal memory encoding, and the Word List Recognition Test to assess verbal memory retrieval all showed similar remarkable improvements in the enriched group relative to the control group (*p* < 0.001, *p* = 0.031, and *p* < 0.001, respectively). Finally, the Geriatric Depression Scale showed a reduction in depression symptoms in enriched patients, but not in controls (*p* < 0.001).

Taken all together, olfactory environmental enrichment may enable the brain to compensate for the neuropathology and restore normal functioning, consistent with the idea that olfactory enrichment could be a formidable and facile treatment option for depression.

## Olfactory loss increases neurological risk

### Olfactory loss and the risk of neurological disorders

Olfactory loss accompanies 68 neurological disorders of which we are aware (Table 1). There are also other medical disorders that are accompanied by olfactory loss, such as diabetes, hypertension, cardiovascular disease, cancer, and obesity; disorders that may well have a neurological component (Bartoshuk, 1990; Liu et al., 2018; Zhang et al., 2019; Roh et al., 2021; Faour et al., 2022).

**TABLE 1:** Neurological disorders associated with olfactory loss.

Neurological disorder	References	*N*	Olfactory test
22q11 deletion syndrome	Sobin et al., 2006	62	UPSIT
Agnosia	Kopala and Clark, 1990	77	UPSIT
Alcoholism	Rupp et al., 2004	60	Sniffin’ Sticks
Alzheimer’s disease	Waldton, 1974	100	Odor identification test
Amyotrophic lateral sclerosis	Viguera et al., 2018	147	UPSIT
Anesthesia cognitive impairment	Zhang et al., 2022	242	Sniffin’ Sticks
Anorexia	Roessner et al., 2005	32	Sniffin’ Sticks
Anxiety	Chen X. et al., 2021	107	Sniffin’ Sticks
Autism	Kinnaird et al., 2020	80	Sniffin’ Sticks
Autoimmune encephalitis	Geran et al., 2019	64	Sniffin’ Sticks
Bardet-Biedl syndrome	Iannaccone et al., 2005	15	UPSIT
Bipolar disorder	Kazour et al., 2020	176	Odor identification, odor discrimination
Blepharospasm	Gamain et al., 2021	34	Sniffin’ Sticks
Cerebral palsy	Nakashima et al., 2019	14	Japanese odor stick identification test
Chagas’ disease	Leon-Sarmiento et al., 2014	120	UPSIT
Childhood maltreatment	Croy et al., 2010	22	Sniffin’ Sticks
Cluster headache	Samancı et al., 2021	57	Sniffin’ Sticks
Corticobasal syndrome	Luzzi et al., 2007	7	Odour Perception and Semantics Battery
COVID-19	Vaira et al., 2020	150	CCCRC olfaction test
Creutzfeldt-Jakob disease	Reuber et al., 2001	1	Unnamed test
Cystic fibrosis	Di Lullo et al., 2020	121	Sniffin’ Sticks
Depression (unipolar)	Eliyan et al., 2021	3546	Sniffin’ Sticks
Down syndrome	Cecchini et al., 2016	56	Sniffin’ Sticks
Dystonia	Marek et al., 2018	198	Sniffin’ Sticks
Epilepsy	Khurshid et al., 2019	912	UPSIT, Sniffin’ Sticks, other tests
Essential tremor	Elhassanien et al., 2021	46	Sniffin’ Sticks
Fibromyalgia	Amital et al., 2014	45	Sniffin’ Sticks
Fragile X syndrome	Juncos et al., 2012	83	UPSIT
Friedreich ataxia	Connelly et al., 2002	35	UPSIT
Frontotemporal dementia	Luzzi et al., 2007	11	Odour perception and semantics battery
Gaucher disease	McNeill et al., 2012	60	UPSIT
HIV/AIDS	Zucco and Ingegneri, 2004	48	UPSIT
Huntington’s disease	Fernandez-Ruiz et al., 2003	162	UPSIT
Idiopathic intracranial hypertension	Bershad et al., 2014	38	UPSIT, Sniffin’ Sticks, other tests
Inflammation	Schubert et al., 2015	1611	San Diego odor identification test
Kallmann syndrome	Gregson and Smith, 1981	26	Campbell Gregson procedure
Lewy body dementia	Yoo et al., 2018	217	Cross-cultural smell identification test
Memory loss with aging	Doty et al., 1984	1995	UPSIT
Migraine headaches	Whiting et al., 2015	100	UPSIT
Mild cognitive impairment	Peters et al., 2003	100	Sniffin’ Sticks
Multiple sclerosis	Atalar et al., 2018	55	CCCRC olfactory test
Multiple-system atrophy	Abele et al., 2003	8	Sniffin’ Sticks
Myasthenia gravis	Leon-Sarmiento et al., 2012	27	Spanish UPSIT
Myotonic dystrophy	Masaoka et al., 2011	7	T&T olfactometer
Narcolepsy	Buskova et al., 2010	66	UPSIT
Niemann-Pick	Mishra et al., 2016	2	Not indicated
OCD	Berlin et al., 2017	30	UPSIT, hedonics and intensity
Parkinson’s disease	Haehner et al., 2009	50	Sniffin’ Sticks
Long covid	Burges Watson et al., 2021	9000	Self-report
Posterior cortical atrophy	Witoonpanich et al., 2013	15	UPSIT
Posttraumatic stress disorder	Vasterling et al., 2000	68	UPSIT
Prenatal alcohol syndrome	Bower et al., 2013	16	San Diego odor identification test
Progressive supranuclear palsy	Shill et al., 2021	281	UPSIT
Pure autonomic failure	Goldstein and Sewell, 2009	51	UPSIT
REM sleep behavior disorder	Iranzo et al., 2021	140	UPSIT
Repetitive head impacts	Alosco et al., 2017	123	Brief smell identification test
Schizophrenia	Kopala et al., 1993	98	UPSIT
Semantic dementia	Luzzi et al., 2007	20	Odor naming
Spinocerebellar ataxias	Abele et al., 2003	24	Sniffin’ Sticks
Stroke	Wehling et al., 2015	78	Sniffin’ Sticks
Systemic lupus erythematosus	Schoenfeld et al., 2009	100	Sniffin’ Sticks
Systemic sclerosis	Amital et al., 2014	65	Sniffin’ Sticks
Tourette syndrome	Kronenbuerger et al., 2018	56	Sniffin’ Sticks
Traumatic brain injury	Frasnelli et al., 2016	63	Sniffin’ Sticks
Usher syndrome	Ribeiro et al., 2016	130	Sniffin’ Sticks
Vascular dementia	Suh et al., 2020	1	Korean Sniffin’ Sticks
Wilson’s disease	Chen L. et al., 2021	50	UPSIT
Zika/Guillain-Barré syndrome	Lazarini et al., 2022	38	Sniffin’ Sticks

While we have drawn attention to the remarkable number of neurological disorders that are correlated with olfactory loss, we don’t know in each case whether these associations are causal, and in most cases, we don’t even know if the olfactory loss comes before or after the onset of the other neurological symptoms. Moreover, we don’t know if there are common mechanisms underlying the olfactory or other neurological symptoms. At the same time, the fact that olfactory enrichment can ameliorate both the olfactory loss and the neurological symptoms of the various disorders with which it has been tested raises the possibility that there is a causal mechanism in which the loss of olfaction increases the risk of expressing the symptoms characteristic of the various disorders. The large number of correlations adds to the weight of evidence suggesting a causal, rather than simply a correlational relationship in some or many of these relationships.

### How can environmental enrichment improve outcomes for neurological disorders including depression?

While environmental enrichment can relieve the symptoms of various neurological disorders that have been imposed on lab animals, that experience does not mend all the damage in the brain, but rather, the brain seems to compensate for the damage under enriched conditions by making a neural work-around that normalizes the cognition and emotions without directly fixing the underlying problem.

## Discussion

### The need for olfactory stimulation

The loss of olfaction in each of these neurological disorders may be due to a common disruption to normal neural mechanisms. But given the very large number of very different neurological disorders with very different etiologies, that possibility seems unlikely. Another possibility is that the olfactory loss precedes the neurological symptoms, which is the case for Alzheimer’s disease, Parkinson’s disease, the cognitive loss that can accompany long COVID, and depression (Walker et al., 2021; Zamponi et al., 2021). Similarly, olfactory loss may precede all the disorders listed above and if olfactory loss precedes the cognitive, emotional, and motor symptoms, perhaps the olfactory loss plays a role in increasing the risk of developing these functional problems. On the other hand, olfactory enrichment may allow the brain to construct workarounds to normalize the cognitive, emotional, and motor issues, as seen in the animal models of human neurological disorders after environmental enrichment.

Indeed, adequate olfactory stimulation may confer a brain/cognitive reserve, that allows the human brain to compensate for neuropathology and avoid experiencing the symptoms typically associated with neurological disorders (Stern et al., 2019). This phenomenon has been reported for Alzheimer’s disease, where some individuals don’t experience the cognitive loss despite the high level of neuropathology (Nilsson and Lövdén, 2018; Montine et al., 2019; Stern et al., 2020). The ability to avoid memory problems may be due to previous intellectual stimulation (Hertzog et al., 2008; Cooper et al., 2017).

It may be that humans in the affluent world are chronically deprived of the high levels of olfactory stimulation that was present when humans evolved. When people have a brain that is chronically deprived of sufficient olfactory stimulation, it may allow the symptoms of neurological disorders to emerge, just as lab animals display the neurological symptoms when they are deprived of sufficient environmental stimulation, but do not show these symptoms when they are living in an enriched environment. In both humans and rodents, the neurological problem remains, but the enriched brain can compensate for that damage and veil the symptoms. Increased vulnerability of older adults to such disorders therefore may be the reason that poor olfactory ability predicts all-cause mortality in humans after middle age, with anosmic individuals experiencing a fourfold increase in mortality risk compared to those with normal olfactory ability (Wilson et al., 2011; Gopinath et al., 2012; Pinto et al., 2014; Devanand et al., 2015; Ekström et al., 2017; Schubert et al., 2017; Fuller-Thomson and Fuller-Thomson, 2019; Kamath and Leff, 2019; Liu et al., 2019; Choi et al., 2021; Pinto, 2021; Xiao et al., 2021; Pang et al., 2022). Ongoing olfactory enrichment may therefore be a critical environmental component needed to prevent the symptoms of neurological disorders, including depression, from being expressed over the course of a lifetime.

## Data availability statement

The original contributions presented in this study are included in the article/supplementary material, further inquiries can be directed to the corresponding author/s.

## Author contributions

Both authors listed have made a substantial, direct, and intellectual contribution to the work, and approved it for publication.
